# Efficient assembly of *de novo *human artificial chromosomes from large genomic loci

**DOI:** 10.1186/1472-6750-5-21

**Published:** 2005-07-05

**Authors:** Joydeep Basu, George Compitello, Gregory Stromberg, Huntington F Willard, Gil Van Bokkelen

**Affiliations:** 1 Institute for Genome Sciences & Policy, Duke University, Durham, NC 27708, USA; 2 Athersys Inc., 3201 Carnegie Avenue, Cleveland, OH 44115, USA

## Abstract

**Background:**

Human Artificial Chromosomes (HACs) are potentially useful vectors for gene transfer studies and for functional annotation of the genome because of their suitability for cloning, manipulating and transferring large segments of the genome. However, development of HACs for the transfer of large genomic loci into mammalian cells has been limited by difficulties in manipulating high-molecular weight DNA, as well as by the low overall frequencies of *de novo *HAC formation. Indeed, to date, only a small number of large (>100 kb) genomic loci have been reported to be successfully packaged into *de novo *HACs.

**Results:**

We have developed novel methodologies to enable efficient assembly of HAC vectors containing any genomic locus of interest. We report here the creation of a novel, bimolecular system based on bacterial artificial chromosomes (BACs) for the construction of HACs incorporating any defined genomic region. We have utilized this vector system to rapidly design, construct and validate multiple *de novo *HACs containing large (100–200 kb) genomic loci including therapeutically significant genes for human growth hormone (HGH), polycystic kidney disease (PKD1) and ß-globin. We report significant differences in the ability of different genomic loci to support *de novo *HAC formation, suggesting possible effects of *cis*-acting genomic elements. Finally, as a proof of principle, we have observed sustained ß-globin gene expression from HACs incorporating the entire 200 kb ß-globin genomic locus for over 90 days in the absence of selection.

**Conclusion:**

Taken together, these results are significant for the development of HAC vector technology, as they enable high-throughput assembly and functional validation of HACs containing any large genomic locus. We have evaluated the impact of different genomic loci on the frequency of HAC formation and identified segments of genomic DNA that appear to facilitate *de novo *HAC formation. These genomic loci may be useful for identifying discrete functional elements that may be incorporated into future generations of HAC vectors.

## Background

Human artificial chromosomes are currently being developed as tools for functional annotation of the genome and as potential vectors for gene therapy and other biotechnological applications (reviewed in [1,2]). Strategies for the creation of artificial or engineered chromosomes can be broadly divided into two classes: *top down*, based on the truncation of an existing chromosome into a much smaller mini-chromosome suitable for further manipulation, and *bottom up*, whereby defined, cloned chromosomal elements are assembled *in vitro *into a prefabricated unit that is capable of nucleating formation of a HAC *de novo *upon introduction into human cells [1-4]. These cloned chromosomal elements may also be assembled in cultured cells through a combination of non-homologous recombination and end-joining mechanisms [5]. Thus far, both approaches have resulted in the creation of a *de novo *HAC composed of large concatamers of the input DNA species (reviewed in [2]). These *de novo *HACs are mitotically stable in the absence of selection, associate with key centromere and kinetochore proteins and are functionally comparable to the native chromosomes of the host cell. Furthermore, HACs containing two genomic loci, for HPRT and GCH1, have demonstrated evidence of functionality in certain cell culture models, establishing the potential application of HACs as vectors for gene transfer [6-8].

Creation of artificial chromosomes *de novo *minimally requires a cloned centromeric element of either natural [9] or synthetic [5] origin. Only higher-order alpha-satellite DNA, found at the centromeres of all normal human chromosomes [10], has been shown to be capable of nucleating centromere formation *de novo*. Alpha-satellite DNA consists of a hierarchical structure of tandem repetitive monomers of ~170 bp, which may be further organized into higher-order repeat units over many hundreds of kilobases [11]. Higher-order alpha-satellite DNA is capable of establishing the assembly of a protein/DNA complex, the kinetochore, which mediates the interactions between the chromosome and the spindle apparatus during cell division [12,13]. These and other lines of evidence suggest that alpha-satellite DNA of this type represents the functional centromere in normal human chromosomes [10,14].

In addition to a functioning centromere, linear artificial chromosomes require synthetic telomeres, which are capable of seeding large telomeric arrays *in vivo *[15]. However, telomeric DNA is not required for the creation of circular HACs *de novo*, and its presence or absence appears to have no significant impact on the stability of such HACs [16].

Finally, HAC vectors require origins of DNA replication that are functionally analogous to Autonomously Replicating Sequence (ARS) elements in yeast [17]. However, mammalian origins of replication remain poorly defined [18], although at least some mammalian origin elements ("replicators") have been documented that continue to behave as such when translocated to an ectopic chromosomal location [19,20]. Notwithstanding uncertainty about the genomic features that constitute an origin, mammalian origins of replication have been shown to occur on average once every ~100 kb [21,22]. *De novo *HAC formation at frequencies of at least 10% has been documented from BAC vectors containing only cloned alpha-satellite DNA [2-4,16], implying that replication origin function must be supplied by elements within alpha-satellite or the BAC vector backbone. Notwithstanding this result, we reasoned that we could augment *de novo *HAC formation by providing any genomic fragment of at least 100 kb in *cis *to the centromeric element of the HAC vector, thereby providing additional origin function and potentially additional unidentified functional elements, or simply by providing improved stability as a consequence of an increase in the size of the HAC vector.

To circumvent technical difficulties in the manipulation of high-molecular weight DNA by traditional cloning techniques [23], we have developed a novel transposition-based approach to rapidly retrofit genomic BAC clones with telomeres and other key functional elements. Ligation of the linearized derivatives of these retrofitted BAC vectors (referred to as "BAC-GEN" vectors) with a complementary linearized BAC vector containing a synthetic D17Z1 alpha-satellite array [5,24] and a telomere (referred to as "BAC-CEN") results in the assembly of a linear prefabricated HAC vector containing the defined genomic fragment of interest. Here, we apply this method to the construction and validation of HAC vectors containing different large fragments from the human genome, representing a diverse group of functionally validated *de novo *HACs containing human genes.

In the course of this work, we observed that certain genomic loci appear to greatly facilitate the formation of *de novo *HACs, suggesting the existence of at least one additional parameter to be optimized during the development of future iterations of HAC vectors. Such loci may contain origins of replication [20,25], scaffold or matrix attachment regions (S/MARs) [26] or other functionally significant chromosomal elements that might contribute to HAC formation and/or stability. We have applied this approach to the construction of a prefabricated HAC vector incorporating the entire 200 kb ß-globin genomic locus, which contains a well-defined mammalian origin of replication [20]. We demonstrate efficient rates of formation of these ß-globin HACs and provide evidence of persistent gene expression. Taken together, the ability to rapidly create multiple, functionally validated BAC-based HAC vectors incorporating any defined genomic locus represents a promising advance in the development of HAC vector technology.

## Results

### Assembly of linear, prefabricated HAC vectors

**Figure 1:**
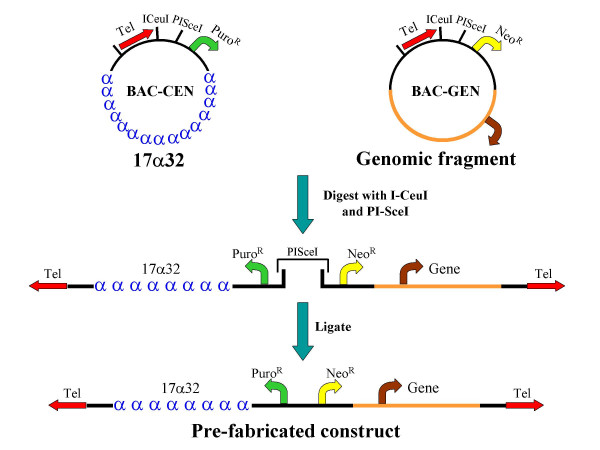
**Strategy for construction of a bimolecular, prefabricated, linear HAC vector. **Digestion of BAC-CEN and BAC-GEN vectors with the ultra-rare homing endonucleases I-CeuI and PI-SceI permits directional ligation of both "arms" to form a linear HAC vector.

The bimolecular BAC-based HAC vector system is comprised of a centromere-containing "CEN arm" containing an 86 kb D17Z1-derived synthetic alpha-satellite array [5] and a "GEN arm", incorporating a defined, large (>100 kb) genomic fragment. Both BAC-CEN and BAC-GEN additionally contain ~800 bp synthetic telomeres [15] and selectable markers as indicated in Figure 1. A linearized CEN arm is generated by digestion of BAC-CEN with the ultra-rare homing endonucleases I-CeuI and PI-SceI, which creates a unique, non-self complementary overhang.

Any genomic BAC vector may be retrofitted to form a BAC-GEN vector by transposition with a custom-built Tn5 based transposon [27] incorporating a telomere, selectable markers and appropriately oriented recognition sites for I-CeuI and PI-SceI [3]. Transposition of the telomere cassette is non-site specific, and insertions into either the BAC vector backbone or genomic insert can be isolated. We were able to generate vector backbone transpositions for all VJ104-based genomic BACs (generated by "shotgun" subcloning, see Methods) and genomic transpositions for BACs containing the HGH, PKD1 and ß-globin loci. For the latter, the integration site of the transposon was established by direct sequencing, and the transposon was confirmed to not interrupt either the target gene or its established regulatory elements (data not shown).

**Figure 2:**
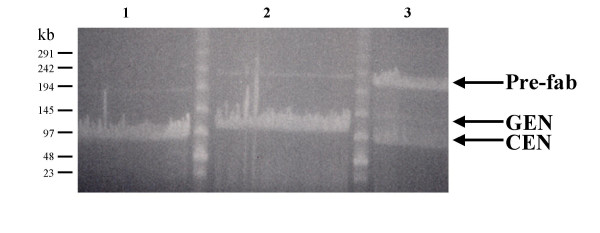
**Pulsed Field Gel Electrophoresis showing creation of HAC vector. **Ligation reactions were set up using linearized BAC-CEN alone (Lane 1), linearized BAC-GEN alone (Lane 2), or linearized BAC-CEN and BAC-GEN together (Lane 3). A clear ligation product (arrow), the linear HAC vector, is only visible in Lane 3. Note that trace amounts of re-circularized CEN and GEN arms are detectable in Lanes 1 and 2, but these do not significantly affect assembly or purification of the HAC.

The BAC-GEN arm is linearized in a similar manner to BAC-CEN, generating an overhang that is complementary only to the residual PI-SceI overhang created from the CEN arm. A ligation between the linearized CEN and GEN arms generates a prefabricated, linear HAC vector (Figs. 1,2), which may be gel-purified and introduced into mammalian cells by transfection or direct nuclear microinjection; alternatively, the entire ligation reaction may be transfected directly.

We assembled a collection of thirteen BAC-GEN vectors representing different genomic loci (100–200 kb) that were shotgun subcloned or identified through the public databases as containing genes of potential therapeutic interest (see Methods). A summary of the sizes and chromosomal origins of each of these genomic fragments is indicated in Table 1. Prefabricated HAC vectors containing each of these genomic loci were generated by the methodology described in Figures 1 and 2 and transfected into HT1080 cells, resulting in the formation of large, cytogenetically visible *de novo *HACs presumably composed of concatamers of the initial DNA species. Assembly of the prefabricated HAC was monitored in all cases by PFGE or FIGE (Fig. 2) (see Methods for additional details).

### Impact of different genomic loci on *de novo *HAC formation

**Figure 3:**
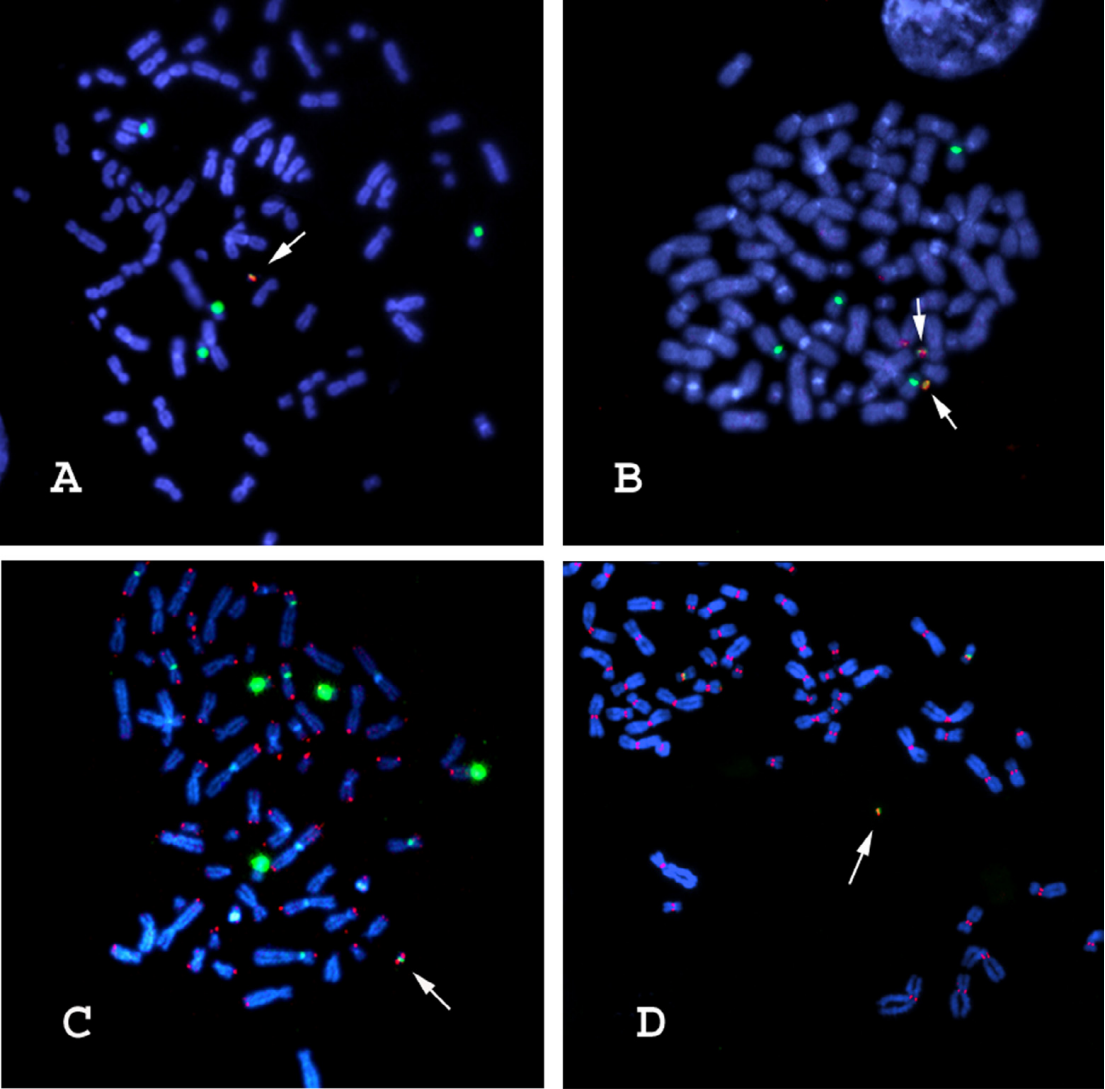
**Cytogenetic validation of *de novo *HAC vectors containing the ß-globin genomic locus. **A) D17Z1 alpha-satellite (green), BAC vector (red). (B) D17Z1 alpha-satellite (green), ß-globin genomic locus (red). (C) D17Z1 alpha-satellite (green), telomere DNA (red). (D) D17Z1 alpha-satellite (green), CENP-C (red). In all cases, DNA is in blue (DAPI). Arrows point to *de novo *HACs.

The efficiency of *de novo *HAC formation from prefabricated HAC vectors containing different genomic loci is summarized in Table 1. All HACs were validated structurally by FISH analysis with probes against D17Z1 alpha-satellite, BAC vector, genomic insert and telomeric DNA, as shown in Figure 3A–C. *De novo *centromere formation was demonstrated by the localization of CENP-C to the HAC (Figure 3D); CENP-C is an established marker of functional centromeres [28,29]. Although the numbers of clones are modest, it is clear from the data in Table 1 that not all the genomic loci examined form HACs at similar efficiency. For example, some genomic loci (e.g. G2, G17 and G19) form HACs only rarely (<10%). In contrast, fragments G6 (75%) and G16 (71%) appear to facilitate *de novo *HAC formation much more efficiently. Fragment G6 is a 101,704 bp NotI fragment represented on the genome scaffold by BAC accession numbers AC004854.2 and AC004847.3. Fragment G16 is a 84,886 bp NotI fragment represented on the genome scaffold by BAC accession numbers AC104698 and AC016907. Other loci, including the HGH and PKD1 genomic loci, form HACs at intermediate frequencies (19% and 21%, respectively). Interestingly, the HGH locus in a prefabricated vector formed HACs at approximately the same frequency as reported earlier for the same locus in a different vector system [3], suggesting that this intermediate frequency is indeed a property of the genomic locus.

### Functionality of ß-globin HAC vectors

**Figure 4:**
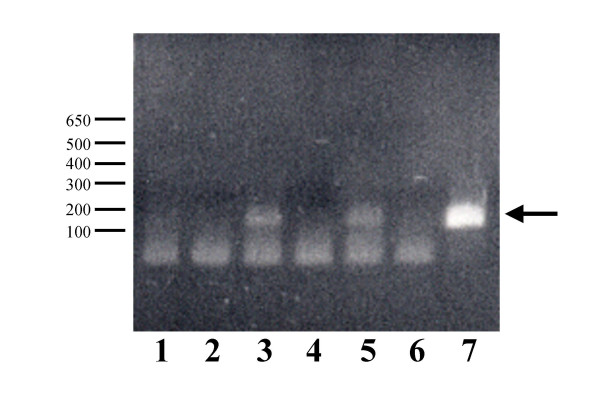
**Analysis of ß-globin mRNA expression from cell lines containing *de novo *ß-globin HACs after 30 days in the absence of selection. **Poly A+ RNA from individual cell clones was subjected to first strand synthesis and PCR with primers specific to exon-3 of the ß-globin gene. Arrow indicates the ß-globin PCR product. 1) Untransfected HT1080. (2) Untransfected HT1080, no reverse transcriptase (RT). (3) ß-globin HAC clone #1, +RT. (4) ß-globin HAC clone #1, -RT. (5) ß-globin HAC clone #2, +RT. (6) ß-globin HAC clone #1, -RT. (7) ß-globin genomic DNA control

It is important to determine whether genes introduced as part of HAC vectors are functional in the recipient host cell. As a proof of principle, we have generated evidence of sustained gene expression from cytogenetically validated HACs containing the entire 200 kb ß-globin genomic locus, establishing the potential application of future iterations of these HACs for gene transfer. As shown in Figure 4, expression from the third exon of ß-globin is continuously detectable by RT-PCR from clones containing ß-globin HACs after 30 days of culture in the absence of selective pressure in the cell line HT1080, a fibrosarcoma line that does not express ß-globin. Expression of ß-globin continues to be detectable in the absence of selection for >90 days of continuous culture (data not shown).

## Discussion

### Design and validation of bimolecular BAC-based HAC vectors

HACs are believed to function by reproducing the three known critical elements of naturally occurring chromosomes: centromeres, telomeres and origins of replication [1,2]. Optimization of HAC formation may theoretically be achieved by the systematic identification and manipulation of factors that affect the efficiency of formation and subsequent stability of each of these key functional elements. For example, in previous studies, we and others have used *de novo *centromere formation as an assay to design and evaluate synthetic D17Z1-based alpha-satellite arrays with modifications in the density and distribution of the consensus CENP-B box, a protein binding site known to impact the effectiveness of *de novo *centromere formation [3,30]. We have shown that D17Z1-based arrays containing an increased number of CENP-B boxes relative to native D17Z1 show a corresponding increase in the efficiency of *de novo *HAC assembly [3].

In this report, we employ *de novo *HAC formation as an assay to identify genomic loci that are highly efficient in HAC formation and are thus candidates for containing origins of replication, S/MARs or other *cis*-acting functional elements that may impact the formation and/or maintenance of HACs. We assembled a collection of genomic DNAs in the 100–200 kb size range (a size range providing a reasonable expectation of containing at least one of these functional units [21]) and assayed their ability to support the formation of *de novo *HACs.

Construction of multiple BAC-based HACs demands the development of novel BAC modification methodologies, owing to the difficulties inherent in the manipulation of high-molecular weight DNAs by traditional subcloning techniques [23]. A first step towards achieving eventual defined composition of matter for HAC vectors [2] requires moving away from uncontrolled *in vivo *mechanisms for HAC vector assembly [5,6,8] towards construction of pre-fabricated HAC vectors containing clearly defined centromeric and other genomic elements. The controlled and systematic generation of large synthetic or naturally derived alpha-satellite arrays is itself difficult, but manageable [3,5,30,31]. Once derived however, these alpha-satellite arrays must be efficiently and predictably brought together with the genomic fragment of interest to create a prefabricated HAC vector.

A number of *in vivo *site-specific recombination approaches to join large alpha satellite arrays with large genomic fragments have been reported [32,33], involving multi-step recombinogenic methodologies whose limitations are evidenced by the fact that few genomic loci have to date been reported to have been successfully incorporated into a HAC vector. In contrast, our transposon-based strategy for the construction of bimolecular BAC-based HAC vectors has facilitated the high-throughput creation of *de novo *HACs from multiple large genomic loci. Additionally, our laboratory has recently reported a complementary transposon-based methodology for the rapid retrofitting of genomic BACs into unimolecular BAC-HAC vectors, by the mobilization of a single transposable element containing alpha-satellite, telomeric DNA and mammalian selectable markers [3]. This latter approach may be even more efficient overall, avoiding the requirement for an *in vitro *ligation between two distinct DNA species and providing the flexibility to create either circular or linear derivatives of the same BAC-HAC vector as needed [3].

On the other hand, the strategy detailed in the current report involves modification of a target genomic BAC with a much smaller transposable element, enabling the retrofitting of the target BAC with the desired functional cassettes to be achieved in a more straightforward and efficient manner. However, we cannot rule out the possibility that trace amounts of recircularized CEN or GEN arms (Figure 2) are co-purified with the prefabricated species and contribute to *de novo *HAC formation, or, if the entire ligation reaction is used, determine to what extent individual, linearized CEN or GEN arms contribute to *de novo *HAC assembly by end-joining or non-homologous recombination mechanisms [5]. Given that the resultant *de novo *HACs are ultimately produced by the uncontrolled concatamerization of the starting DNA species, this point, while noteworthy, is not significant in our view.

It is important to view the current strategy attempting to create prefabricated HAC vectors in the context of iterative progress towards the eventual achievement of defined composition of matter for *de novo *HACs, while understanding that this remains a goal yet to be accomplished [2]. Only when this objective has been reached will dissection of the relative contributions of "contaminating" alternative forms of the starting vectors be truly meaningful. Nevertheless, the overall effectiveness of the current methodology has facilitated the construction and functional validation of multiple *de novo *HACs derived from a significant collection of genomic loci, thereby establishing HAC technology as a general one suitable for analysis of in principle any locus in the genome.

### *cis*-acting genomic loci affect *de novo *HAC formation

Although the current study was initiated to provide a functional platform for the identification of functional genomic elements in a manner analogous to that first used to identify Autonomously Replicating Sequences (ARS elements) in yeast [17], we stress that we currently have no independent biochemical or other confirmation that the observed effects on HAC formation frequencies are actually related to the presence or absence of replication origins, S/MARs or any other specific functional elements. Nevertheless, it is not unreasonable to propose variation in origin function as one hypothesis to explain the observed differences in *de novo *HAC assembly, and further experiments will be required to explore this possibility.

As seen in Table 1, the majority of genomic fragments surveyed support *de novo *HAC formation at frequencies consistent with previous reports using vectors that lack genomic fragments or contain a limited number of other genomic fragments [3,4,16,34]. Indeed, our own previous results using BAC vectors containing only the synthetic, 86 kb D17Z1-based alpha-satellite array used in the current report generates a baseline for *de novo *HAC formation of 10.5% from 38 analyzed clones [3]. The majority of genomic fragments surveyed (G2, G8, G10, G14, G17, G19) appear to support *de novo *HAC formation at frequencies comparable to alpha-satellite alone [3]. We note that the unimolecular BAC-HAC vector reported in [3] incorporating the same HGH genomic region used in the current report forms *de novo *HACs at similar intermediate frequencies comparable to that observed here (15% in [3], 19% in the current report). Other genomic loci (G4, G6, G11, G16 and the ß-globin locus) do appear to facilitate *de novo *HAC formation at efficiencies above the baseline (see Table 1). Most notably, genomic loci G6 and G16 support *de novo *HAC formation at frequencies of over 70%, substantially higher than other genomic fragments of similar size. These effects clearly cannot be explained as the result of a simple increase in the size of the HAC vector, as this would result in a general increase in *de novo *HAC formation regardless of which specific genomic fragment was utilized. Further dissection of these 100 kb fragments is currently underway to isolate smaller subfragments that may be incorporated as a functional cassette into the design of future iterations of HAC vectors.

### Cell line specific effects on *de novo *HAC formation and gene expression

The current report is based on the analysis of *de novo *HAC formation in the HT1080 fibrosarcoma cell line, consistent with all previously reported studies on the assembly of *de novo *HACs (reviewed in [1,2]). Although no systematic examination of the role of the host cell environment on rates of *de novo *HAC formation has yet been reported, it remains formally possible that the *cis*-acting effects of adjacent genomic loci on *de novo *HAC formation are contingent on certain cellular environments. Although the choice of the HT1080 cell line for use in this and other related studies [1,2] is largely historical, we have observed comparable rates of *de novo *HAC formation in the 293 and other closely related cell lines using BAC vectors containing only cloned alpha-satellite DNA (our unpublished observations).

The observation that *de novo *HACs incorporating a 216 kb ß-globin genomic locus do in fact express ß-globin in the non-erythroid HT1080 cell line is noteworthy (Figure 4). Although these HACs contain the entire 5' and 3' Locus Control Regions established as being critical for the regulation of globin gene expression in a physiologically appropriate manner [37], it appears that the cloned ß-globin genomic DNA, upon introduction into the cell nucleus in the context of a HAC vector, does not adopt the repressive chromatin configuration found at the endogenous, host cell ß-globin locus. This observation may potentially be highly significant if found to be consistent with the behavior of additional genes upon introduction into the nucleus as HAC vectors, as it impacts on the ability to reproducibly and reliably obtain cell- and tissue specific-patterns of gene expression for applications in biotechnology. Finally, although we have not rigorously quantified ß-globin gene expression from clones containing *de novo *ß-globin HAC vectors over time, we do note that ß-globin gene expression is stably observed by the RT-PCR assay of Figure 4 over the 90-day time period used in the current report (data not shown).

## Conclusion

HAC vectors provide a novel approach to human genome annotation and gene transfer that may ultimately circumvent many of the technical difficulties currently associated with standard, retroviral-based gene therapy vectors [2]. These include position effects on gene expression and transgene silencing (reviewed in [35]) and a strict, upper packaging limit permitting delivery of only about 8 kb of foreign DNA [36], precluding the incorporation of critical genomic elements that may be required for physiologically meaningful expression of a therapeutic transgene [37]. Additionally, integration of viral vectors into the host genome has been shown to result in oncogene activation leading to cancer [38]. Finally, viral vectors used in recent clinical trials have resulted in severe immunological reactions and death [39].

In contrast to the capacity limits of conventional gene transfer vectors, we have been able to design and construct HAC vectors that contain the entire 200 kb ß-globin genomic region, including the 5' Locus Control Region required for ß-globin gene regulation and expression [37], thereby bypassing the requirement to identify, dissect and repackage critical regulatory elements into mini-genes capable of being delivered by potentially immunogenic viral vectors. We have shown sustained gene expression from these ß-globin HACs in the absence of selective pressure during at least 90 days of continuous culture. Similarly, we have designed and fabricated HAC vectors incorporating the entire HGH (159 kb) and PKD1 (208 kb) genomic loci; the PKD1 cDNA itself is over 14 kb, well outside the range of retroviral gene therapy vectors [40].

In summary, we anticipate that the functional identification and optimization of individual chromosomal components using the HAC vector systems described here and elsewhere [2,3] will eventually permit the design and construction of prefabricated, custom built HAC vectors incorporating any therapeutic gene in the context of its full complement of endogenous, genomic regulatory elements. HAC vectors may therefore not only fulfill their potential in biotechnology, but will additionally lead to significant advances in the functional annotation of the genome.

## Methods

### Construction of BAC-CEN and BAC-GEN

BAC-CEN is a derivative of pBAC108L, modified to include ~800 bp of synthetic telomeric sequence (made as described in [5]), a puromycin resistance cassette and an adapter containing the recognition sites for the homing endonucleases I-CeuI and PI-SceI (New England Biolabs). 86 kb of synthetic, D17Z1-based alpha-satellite DNA (representing 32 tandem copies of the 2.7 kb higher-order repeat) was subcloned as a BamHI-BglII fragment into a unique BamHI site on BAC-CEN, to create BAC-CEN17a32.

Individual BAC-GEN vectors were generated by transposon-mediated retrofitting of defined, genomic BACs [27]. To create the transposon targeting vector, the EZ:TN transposon (Epicentre Technologies, Madison WI) was modified to include ~800 bp of synthetic telomeric DNA, a neomycin/kanamycin resistance marker and an adapter containing the recognition sites for the homing endonucleases I-CeuI and PI-SceI, as described above. Transposition reactions were carried out as recommended by the manufacturer. Target genomic BACs were identified and procured through the genome project databases (PKD1: CTD2517G10-208 kb, ß-globin: CTD264317-216 kb, HGH: CTD2202F23-159 kb, all obtained from Research Genetics), or were created by "shotgun" subcloning of size-selected, NotI-digested whole genomic DNA into the BAC vector VJ104, a pBAC108L derivative [5]. Transposition of the telomeric unit into the vector backbone of a genomic BAC was identified as an upward shift in the electrophoretic mobility of the corresponding vector band upon digestion with NotI (data not shown).

### Preparation of the prefabricated HAC vector

10 µg (equimolar amounts) of each of BAC-CEN and the selected BAC-GEN DNAs were mixed together into a single 1.5 ml eppendorf tube and digested with PI-SceI and I-ceuI in a total volume of 200 µl for 3 hours. The homing endonucleases were heat inactivated, and ATP (Epicentre) was added to a final concentration of 1 mM. The linearized CEN and GEN arms were ligated together overnight at room temperature by addition of T4 DNA Ligase (New England Biolabs). In all cases, the assembly of the prefabricated HAC vector was monitored by resolution of the individual species within the ligation reaction using Pulsed Field Gel Electrophoresis (PFGE) (Bio-rad, DR-III), or Field Inversion Gel Electrophoresis (FIGE) (Bio-rad) and confirmed to have proceeded efficiently as shown in Figure 2. Only ligation reactions showing efficient assembly of the prefabricated HAC vector were used for transfections. The ligation product representing the prefabricated vector species could then be gel purified by electroelution of the target band out of the gel slice into 0.5X TBE. The electroeluted DNA species was then dialyzed into ddH_2_O and concentrated into the smallest possible volume using a Microcon YM-100 spin column (Amicon) according to the manufacturer's instructions. The concentrated HAC vector was used directly for transfection as described below. In some cases, the ligation reaction was transfected directly without additional gel-purification of the prefabricated species.

### Cell transfection

Human fibrosarcoma HT1080 cells were transfected using the Fugene-6 (Roche) reagent according to the manufacturer's instructions, and stable clones identified through resistance to puromycin at 3 µg/ml and neomycin at 600 µg/ml. Clones appeared after 7–10 days and were subsequently expanded to generate clonal lines for further analysis. Multiple independent transfections were performed for all 13 HAC species, and the data pooled to generate Table 1.

### Cytogenetic analysis

FISH analysis of clonal lines was carried out according to established procedures. Briefly, a D17Z1-specific alpha-satellite probe was used to initially identify putative HACs. Further structural confirmation of all putative HACs was done by FISH with probes specific to the BAC vector backbone, the genomic insert and telomeric DNA. In all cases, probes were labeled by nick translation in the prescence of Spectrum Green or Spectrum Orange conjugated dUTP (Vysis). For immunofluorescence analysis, slides were immunoreacted with a rabbit anti-CENP-C antibody [5] at a concentration of 1/2000 in PBS and detected with FITC conjugated goat anti-rabbit IgG (Molecular Probes). Images were acquired through a Zeiss fluorescence microscope and CCD camera. Clones containing cytogenetically confirmed HACs were further validated by additional cytogenetic examination following continuous culture in the absence of selection for at least 90 days (data not shown).

### Gene expression

RT-PCR reactions to assay ß-globin gene expression were carried out using the ExpressDirect system (Pierce) according to the manufacturer's instructions. RNA was prepared from HT1080 cells and HAC-containing clones, using standard techniques.

## List of abbreviations

BAC- Bacterial artificial chromosome

HAC- Human artificial chromosome

ARS- Autonomously replicating sequence

CENP- Centromere protein

HPRT- Hypoxanthine guanine phosphoribosyltransferase

S/MARs- Scaffold/matrix attachment regions

GCH1- Guanosine triphosphate cyclohydrolase 1

PFGE- Pulsed Field Gel Electrophoresis

FIGE- Field Inversion Gel Electrophoresis

FISH – Fluorescence in situ hybridization

## Authors' contributions

JB designed and constructed the vectors, planned and coordinated the experiments and wrote the manuscript. GS and GC contributed to vector construction and executed the experiments. GVB and HFW provided critical intellectual feedback and assisted in writing the manuscript.

## Figures and Tables

**Table 1:** Influence of different genomic loci on *de novo *HAC formation

**Genomic Locus**	**Size (kb)**	**Location**	**No. Transfections**	**Freq. HAC formation**^1^
G2	100	9q33	12	1/21 (5%)
G4	98	8q24.3	10	8/19 (42%)
G6	90	7p13	6	9/12 (75%)
G8	125	15q23	5	1/8 (12%)
G10	125	3p16	9	3/16 (19%)
G11	100	22q11.4	11	9/33 (27%)
G14	125	1q32	2	3/14 (21%)
G16	97	2p23	5	5/7 (71%)
G17	97	4q22	8	2/19 (10%)
G19	95	11p13	6	1/26 (4%)
ß-globin	216	11p15.5	6	7/27 (26%)
HGH	159	17q24.2	8	3/16 (19%)
PKD1	208	16p13.3	10	4/19 (21%)

**1: **HAC formation frequency is listed as (# clones containing HAC functionally validated by cytogenetic analysis and mitotic stability for at least 90 days)/(Total # clones screened from all transfections with the genomic locus of interest)
